# Data innovation in response to COVID-19 in Somalia: application of a syndromic case definition and rapid mortality assessment method

**DOI:** 10.1080/16549716.2021.1983106

**Published:** 2022-04-04

**Authors:** Andrew Seal, Mohamed Jelle, Balint Nemeth, Mohamed Yusuf Hassan, Dek Abdi Farah, Faith Mueni Musili, George Samuel Asol, Carlos Grijalva-Eternod, Edward Fottrell

**Affiliations:** a UCL Institute for Global Health, London; b Evidence for Change (E4c), Brussels, Belgium; c Norwegian Refugee Council, Oslo, Norway; d Norwegian Refugee Council, Nairobi, Kenya

**Keywords:** Somalia, Covid-19, surveillance system, mortality, syndromic case definition

## Abstract

**Background:**

During the COVID-19 pandemic, the importance of reliable public health data has been highlighted, as well as the multiple challenges in collecting it, especially in low income and conflict-affected countries. Somalia reported its first confirmed case of COVID-19 on 16 March 2020 and has experienced fluctuating infection levels since then.

**Objectives:**

To monitor the impact of COVID-19 on beneficiaries of a long-term cash transfer programme in Somalia and assess the utility of a syndromic score case definition and rapid mortality surveillance tool.

**Methods:**

Five rounds of telephone interviews were conducted from June 2020 – April 2021 with 1,046–1,565 households participating in a cash transfer programme. The incidence of COVID-19 symptoms and all-cause mortality were recorded. Carers of the deceased were interviewed a second time using a rapid verbal autopsy questionnaire to determine symptoms preceding death. Data were recorded on mobile devices and analysed using COVID Rapid Mortality Surveillance (CRMS) software and R.

**Results:**

The syndromic score case definition identified suspected symptomatic cases that were initially confined to urban areas but then spread widely throughout Somalia. During the first wave, the peak syndromic case rate (311 cases/million people/day) was 159 times higher than the average laboratory confirmed case rate reported by WHO for the same period. Suspected COVID-19 deaths peaked at 14.3 deaths/million people/day, several weeks after the syndromic case rate. Crude and under-five death rates did not cross the respective emergency humanitarian thresholds (1 and 2 deaths/10,000 people/day).

**Conclusion:**

Use of telephone interviews to collect data on the evolution of COVID-19 outbreaks is a useful additional approach that can complement laboratory testing and mortality data from the health system. Further work to validate the syndromic score case definition and CRMS is justified.

## Background

The COVID-19 pandemic has sharpened global focus, both on the need for better health data, and the multiple challenges in collecting it. There has been reduced access to some of the populations most in need of health care, leading to less reliable information on which to base public health decision-making. But the erection of barriers associated with the pandemic has also led to flexibility and innovation amongst health and humanitarian actors.

Data from the government in Mogadishu indicate that the first confirmed case of COVID-19 was found in Somalia on 16 March 2020 [1]. As of 1 May 2021, there had been 14,199 laboratory confirmed cases and the epidemic curve has fluctuated with repeated waves of infection. However, the testing rate in Somalia is very low with less than 5,000 tests currently being conducted per week (equivalent to <0.5 tests/1,000 people/week) with a positive proportion of 9% [2]. Hospital reports of serious COVID-19 morbidity and related deaths are likely to be very low compared to actual levels as the availability and utilization of health services is generally poor. This makes interpretation of the available test results problematic and tracking the progress of the epidemic difficult.

The problem of a low testing rate is common in Africa despite the efforts of Africa CDC and others [3]. The low availability of COVID-19 testing increases the urgency to develop an optimal case definition based on clinical signs and symptoms. As community surveillance is best done remotely by mobile phone, to avoid the risk of transmission and to allow access to remote or insecure regions, the case definition needs to rely on self or lay-reported symptoms.

WHO has published case definitions for suspected, probable, and confirmed cases, and provided guidance on surveillance strategies that have been updated during the course of the pandemic [4,5]. In line with recent global guidance, previous work in Somalia has indicated the predictive importance of the loss or change in taste or smell as a symptom of SARS-CoV-2 infection [6]. Based on this, a clinical case definition has been proposed and applied in an internet survey of behaviours and suspected infections [7,8]. Rapid methods have also been proposed for estimating mortality caused by COVID-19 in situations where laboratory testing has not been available before death and post-mortems are not performed. These include the COVID-19 Rapid Mortality Surveillance verbal autopsy approach developed by the late Peter Byass and colleagues [9].

During the pandemic, the use of telephone-based surveys has scaled up rapidly at a global level. For example, the World Bank has been collecting data on the impacts of COVID-19 from selected African countries using high-frequency phone surveys [10].

The BRCiS NGO – Building Resilient Communities in Somalia consortium, led by Norwegian Refugee Council, has been implementing a long-term cash distribution and community resilience programme in Somalia. Prior to the onset of the COVID-19 pandemic, routine monitoring of BRCiS programme activities was done using regular telephone interviews. This paper describes the rapid adaptation of the existing monitoring and evaluation system and experiences in applying a symptom score-based COVID-19 case definition and the COVID-19 Rapid Mortality Surveillance (CRMS) method to estimate spatial-temporal trends in infection, crude mortality rates and COVID-19 related mortality.

## Methods

### Setting

The BRCiS Safety Net cash transfer programme served 43 communities across 10 regions in Somalia: Banadir, Bari, Bay, Galgadud, Gedo, Hiran, Lower Juba, Lower Shabelle, Mudug, and Sool. It reached 3,048 households in total. Participant households have diverse livelihoods and include pastoralists, agro-pastoralists, IDPs, and urban residents. Employing a Community-Based Targeting approach, the participant households were selected based on a set of vulnerability criteria including household income, assets, disability, and the number of household children under the age of 5 years. Programme participants benefitted from a monthly household cash transfer of $US 20.00 for 2 years. There are also a number of community-level interventions designed to improve livelihoods and resilience against natural disasters [11].

Prior to the onset of the pandemic, telephone interviews were conducted periodically with a sample of beneficiaries for post-distribution monitoring (PDM) purposes. Participants were asked if they had received the intended transfer, how the cash was being used, and what was the status of their household food security. With the outbreak of COVID-19 in Somalia we moved to adapt the PDM approach to track the epidemic impacts on household behaviours, health, and mortality. To achieve these objectives, we enumerated all household members and created a longitudinal database to track mortality. We also added questions on attitudes to COVID-19 and behaviours associated with transmission risk (results will be reported elsewhere), and common COVID-19 symptoms.

### Sampling

The monitoring system used a sample of approximately 1,500 households that contained around 7,000 individuals. The households were randomly selected from the list of current beneficiaries of the BRCiS Safety Net cash transfer programme. Between data collection rounds 3 and 4, the cash distribution programme was enlarged and new communities were registered. During round 4, these additional households were enrolled in the study and enumerated. The individuals from these households then contributed person days of observation to the results reported for round 5.

### Data capture

From June 2020, a team of 15 enumerators, closely supported and supervised by consortium technical staff, periodically collected data from the selected households by conducting telephone interviews with a household respondent, usually the household head. During the interview, the data was entered into a second mobile phone running Open Data Kit Collect. After completion of the interview, the data was uploaded to a server run by ONA Systems where it was compiled into a downloadable database. The interval between the data collection rounds was seven to 8 weeks up to round 4 with 16 weeks in between round 4 and 5.

The average duration of interviews varied by round due to the phase of data collection and adaptation of the questionnaires. Full enumeration of the households increased the interview duration of the interview in round 2 and some data collection rounds included additional questions on household food security, knowledge and perceptions about COVID-19, and attitudes to vaccination (findings to be reported elsewhere). During round 5 the mean duration was 36 minutes.

The enumerators were selected based on their previous experience in public health surveys and familiarity with conducting telephone interviews with vulnerable populations. An initial training session was held plus regular refresher trainings before each round of data collection. Additionally, for each round, mock interviews were conducted to check if enumerators were adhering to the pre-agreed survey protocol, which allowed the monitoring team to spot and address compliance issues early on before the start of data collection.

Data were gathered on deaths, births and in and out movements of household members since the previous interview. Recall periods and days of exposure were calculated individually for each household member depending on the dates when they were interviewed. In rounds 1 and 2 a one-month recall period was used. In round 3 and 4 the recall period was the number of days since the previous interview. In round 5, due to the longer period since the previous data collection round, we used a 3 month recall period beginning on 1 January 2021. If a death was reported within a household the carers of the deceased were interviewed again on a separate occasion to confirm the date and location of death, and determine symptoms preceding death. These Rapid Mortality Surveillance (CRMS) interviews were conducted by a small team of interviewers who had received additional training on use of the questionnaire and how to interview the carers of the deceased in a respectful way.

Real-time data quality checks included the monitoring of interview duration, completeness, and non-response patterns. This was done through a live R shiny dashboard (https://shiny.rstudio.com/gallery/), which provided daily feedback on the performance of enumerators along with the quality of data collected. Additional data checks and cleaning were performed at the analysis stage.

### Analysis

We developed a COVID-19 syndromic scoring approach based on the COVID-19 Rapid Mortality Surveillance (CRMS) tool to estimate the prevalence of COVID-19 symptoms and identify suspected symptomatic cases among household members. The symptoms, and the occurrence of a positive laboratory test, were recorded and then scored as listed in Table 1. The case definition for a suspected case of COVID-19 was a syndromic score of ≥2.0. This case definition was used to estimate the period prevalence of suspected symptomatic COVID-19 and the COVID-19 infection rate, as cases per 1,000,000 per day. We aim to validate this syndromic score case definition (SSCD) approach when adequate testing capacity is available within the country.

**Table 1.** Syndromic scoring system

Symptom or test	Scores
Loss or change in taste or smell	0.9
Cough	0.7
Fever	0.7
Shortness of breath or difficulty breathing	0.6
Fatigue	0.4
Sore throat	0
Headache	0
Diarrhoea or stomach pains	0
Body aches	0
Other	0
Positive laboratory test for COVID-19	2
**Maximum possible score**	**5.3**

A cut-off score of ≥ 2.0 was used to define a suspected case.

To ascertain the likely cause of death, we used the CRMS questionnaire and analysis software, developed in association with WHO and partners, to estimate the likelihood that any death was COVID-19 related. Based on the InterVA method for verbal autopsy interpretation [12], the CRMS analysis method applies Bayesian reasoning and uses approximate prior probabilities of the unconditional likelihood of a COVID-19 death and the likelihood of signs, symptoms, and indicators being reported in relation to a COVID-19 death. The method then calculates the probability of a death being COVID-19 related based on the signs, symptoms and indicators reported by respondents during a verbal autopsy interview. Each death is assigned a probability of being due to COVID-19 or due to other causes. As detailed in the CRMS user guide (available at http://www.byass.uk/interva/crms), the interpretation of the percentage probabilities for deaths from COVID-19 or other causes is a matter for users to decide locally. We classified any death with a COVID-19 probability of 50% or greater as being due to COVID-19. In a separate sensitivity analysis, we compared COVID-19 mortality patterns using probability thresholds at 10% incremental increases from 50% to 90%, as well as COVID-19-specific mortality fractions derived by dividing the sum of the probability of COVID-19 among all deaths by the total number of deaths (see Supplementary file (web Figure W2)).

## Mapping

**Figure 1.**
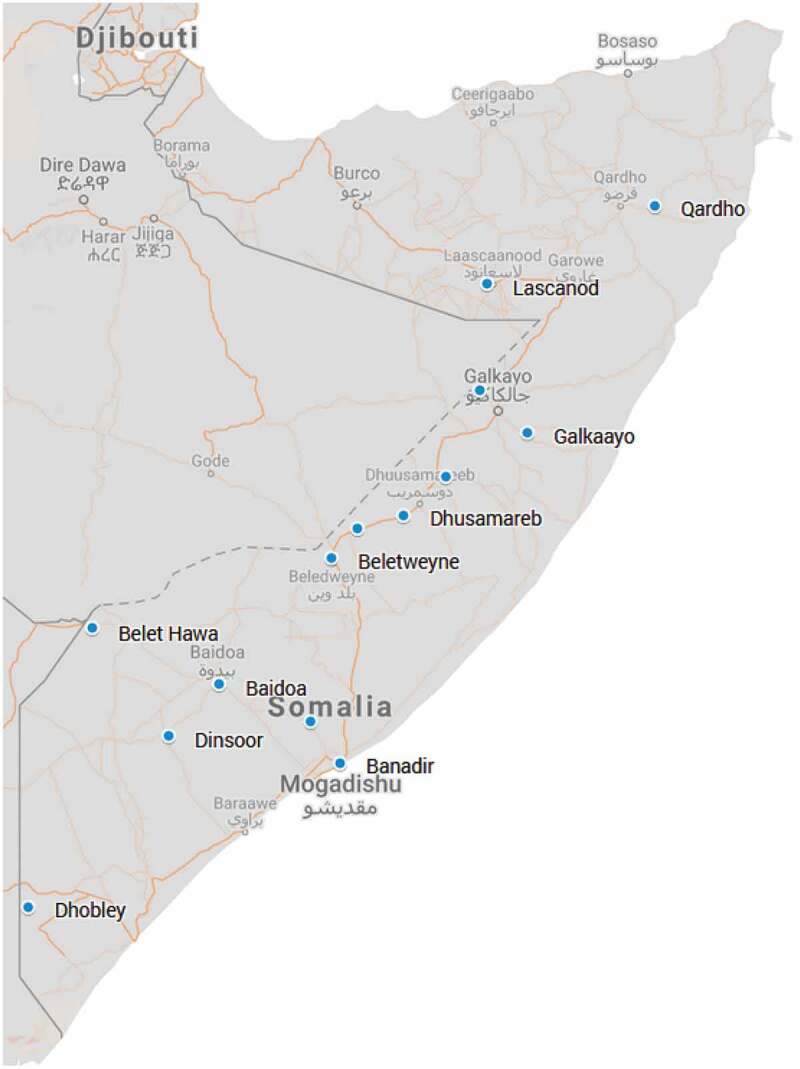
The distribution of participant households by district^1^.

^1^ District locations are shown with a blue dot

To visualise the distribution of the sampling sites, suspected cases, and deaths, community location coordinates were compiled from previously collected project data. To ensure anonymity, the 43 individual community locations were collapsed into their 14 districts for display purposes. Coordinate data was entered into .csv files and imported into Google Earth Pro. After cross-checking the district locations against other published sources, the coordinates were exported as a .kml file and imported into Google My Maps to create the map shown in Figure 1.

## Comparison with laboratory confirmed cases published by WHO

Data on the number of laboratory-confirmed COVID-19 cases was obtained from the WHO global data portal at https://covid19.who.int/. Data on the number of tests conducted in Somalia and the positive proportion was obtained from weekly update reports published by WHO and the Mogadishu Ministry of Health [2]. Data was compiled and graphed in Excel 2019.

## Results

The sample achieved in each data collection round and its characteristics are shown in Table 2. The target sample varied from round to round due some small administrative changes to the beneficiary lists and later on, to an increase in the scope of the safety net intervention, which was scaled up to additional locations in response to changing humanitarian needs. The decision to scale up the programme in specific areas was taken by the implementing partners and linked to recent waves of locust infestation and the COVID-19 pandemic.

**Table 2.** Incidence of suspected COVID-19 infections in safety net beneficiaries

Measurement period	Round 1^1^ Interview respondents only	Round 2	Round 3	Round 4	Round 5
		All household members			
Data Collection	22 Jun–15 Jul	10 Aug–3 Sep	6 Oct–22 Oct	30 Nov–20 Dec	27 Mar–18 Apr
Households included in sample	1,117	1,046	1,115	1,565	1,550
Households interviewed	952	942	947	1,430	1,441
Follow up (%)	85%	90%	85%	91%	93%
Household members included in symptom assessment^1^	952	7,381	6,916	7,418	11,541
Sex (% female)	61.0%	51.4%	51.5%	51.8%	52.2%
HH members with symptomatic COVID-19 in recall period^2^	9	64	36	12	81
Period prevalence of symptomatic COVID-19	0.9%	0.9%	0.5%	0.2%	0.7%
Symptomatic COVID-19 infection rate (cases/1,000,000/day)	311	285	103	34	78

^1^In round 1 only the household respondent was asked about symptoms; in round 2 onwards all household members were included.

^2^In rounds 1 and 2 a one month recall period was used. In round 3 and 4 the recall period was the number of days since the previous interview. In round 5, it was the first 3 months of 2021.

The response rate achieved for each round varied from 85% to 93%. Phones switched off, low batteries, or poor reception were anecdotally reported as the main reasons for non-response. The number of refusals was very low with less than 6 refusals reported per round.

The majority of respondents were female, and females comprised just over half of all household members. The age of the household head ranged from 16 to 95 years (median 40).

The distribution of the surveillance system sample is shown in Figure 1. Suspected cases detected in the first round of data collection were exclusively urban and located in Banadir and Hiran. In subsequent rounds, the suspected cases were geographically much more widely distributed and located in rural as well as urban settlements with a wider range of livelihoods.

**Figure 2.**
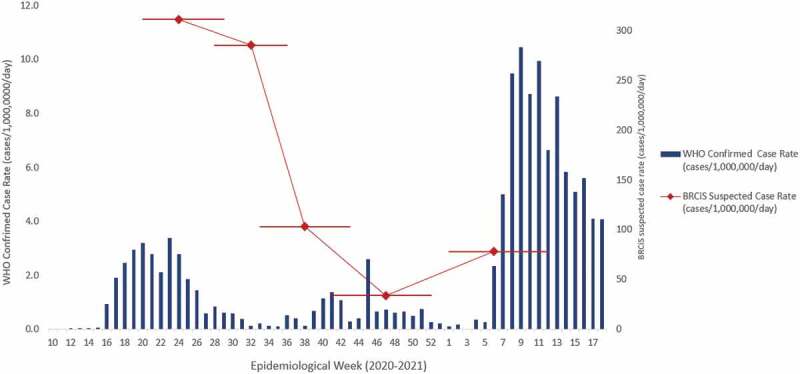
Comparison of laboratory confirmed cases published by WHO for Somalia and suspected symptomatic cases detected by the BRCiS monitoring system^1^.

^1^The horizontal bars indicate the recall periods for each round of data collection. In the first two rounds, a one-month recall period was used, contiguous recall periods were used in rounds 3 and 4, and a 3 month recall period (Jan–Mar) was used in round 5.

The trend in the suspected COVID-19 infection rate is shown in Figure 2, along with the laboratory confirmed cases reported by WHO for the whole of Somalia [13]. During the ‘first wave’, PCR confirmed infections peaked in May and June 2020. Case numbers fell sharply but then rose again with a smaller peak in September before declining once more. In early 2021, cases climbed steeply and reached a much higher peak than seen in the first wave. By week 9 the national case rate had reached 10.5 cases/1,000,000/day.

When interpreting trends in the confirmed case rate, it is important to also consider the testing rate and % positivity. The testing rate in Somalia has remained very low throughout the pandemic but has increased steadily, with a 7-fold increase when comparing the first 12 weeks of the outbreak with the most recent 12 weeks (see Supplementary file (web Figure W1)). Test positivity was extremely high during the early stages of the outbreak but fell sharply and only rose to a maximum of 15% during the second wave in 2021. Taken together, these data suggest that the higher case numbers confirmed during the second wave may be partially attributable to higher levels of testing, and that there was a relative under-detection of cases in wave 1 compared to wave 2.

The suspected infection rate determined using our syndromic case definition peaked during the first round of data collection covering May and June 2020, fell sharply to a minimum from October to November and then rose again at the start of 2021. The general pattern is consistent with the data on lab-confirmed cases. The magnitude is however, as expected, much greater, with a peak syndromic suspected case rate of 311/1,000,000/day compared to a confirmed case rate peak of 11/1,000,000/day. Overall, so far, the suspected syndromic score case rate has been, on average, 99 times higher than the confirmed case rate.

The age and sex profile of people with suspected COVID-19 identified using our syndromic score case definition is shown in Table 3. In general, a higher attack rate is seen in the over 50s, with a higher proportion of females effected. However, a higher than expected proportion is seen in the under-fives.

**Table 3.** Characteristics of participants with suspected COVID-19

Measurement period	Round 2		Round 3		Round 4		Round 5	
	Cases (N)	% Affected	Cases (N)	% Affected	Cases (N)	% Affected	Cases (N)	% Affected
Total cases	64	0.9	36	0.5	12	0.2	81	0.7
Age (years)								
< 5	10	0.7	8	0.6	2	0.1	26	1.2
5–17	22	0.7	16	0.5	3	0.1	29	0.6
18–49	25	1.3	9	0.5	4	0.2	12	0.4
50–69	4	0.9	1	0.2	3	0.7	8	1.1
≥ 70	3	1.3	2	0.9	0	0	6	1.4
Sex								
Male	27	0.7	18	0.5	2	0.1	28	0.5
Female	37	1.0	18	0.5	10	0.3	53	0.9

The crude and under-five mortality rates, measured in the surveillance system, increased during the first wave of infections in Somalia and then fell (Table 4). No increase in deaths were detected in round 5 of data collection. The cause-specific COVID-19 mortality rate, estimated using the CRMS approach, showed a similar pattern with larger increases and decreases. As deaths lag behind infection by about 3 weeks, an increase in suspected COVID-19 deaths may be expected during the next round of data collection.

**Table 4.** Crude, under-5 death, and cause specific death rates

	Round 1	Round 2	Round 3	Round 4	Round 5^1^
Persons under observation	7,618	7,395	6,917	7,418	11,541
Average recall period (days)	70	52	50	56	118
Person days of observation	528,664	349,942	348,870	350,982	1,281,809
Total deaths reported	16	12	17	10	23
Deaths in children <5 years	4	4	4	3	6
Deaths due to suspected COVID-19	5	4	5	4	8
*Cause specific COVID-19 death rate (deaths/million/day)*	9.5	11.4	14.3	11.4	6.2
*Crude Death Rate^2^ (CDR)* *deaths/10,000/day*	0.30	0.34	0.49	0.28	0.18
*Under Five Death Rate^3^ (U5DR) deaths/10,000/day*	_	0.61	0.61	0.46	0.25

^1^In round 5 the families of 2 of the deceased could not be traced and no verbal autopsy was done.

The counts, proportions, and the rate of COVID-19 mortality derived from sensitivity analysis using varying probability thresholds for classification of COVID-deaths, or using the population COVID-specific mortality fractions, are shown in see Supplementary file (web Figure W2). These data show a decrease in COVID-related mortality as the threshold is increased, particularly above 70%. Using the COVID-specific mortality fractions gave very similar results to those obtained using the 50% probability threshold.

## Discussion

This study of the BRCiS COVID-19 monitoring system shows that periodic collection of data via telephone interviews is possible in the Somalia context and has many advantages. Data collection from a cohort of pre-existing cash distribution beneficiaries was rapidly established, and the standard monitoring and evaluation questionnaire was quickly adapted to collect relevant data. As well as allowing rapid deployment of COVID-19 monitoring, utilising a list of cash programme beneficiaries also meant that households from lower SES groups were predominantly included. Based on data from other contexts, households with a lower SES may be expected to be at higher risk of COVID-19 infection and its consequences, making them a useful sentinel population group. Using this sampling approach may help to improve equity by off-setting laboratory testing data that may be more likely to come from urban and higher income groups.

The availability of the CRMS questionnaire and analysis software allowed for the introduction of verbal autopsy and tracking of suspected COVID-19 deaths [9]. It also inspired the creation of our novel syndromic score case definition (SSCD) to monitor the incidence and distribution of suspected cases. The population suspected case rate estimated using the SSCD was much higher than the laboratory test confirmed case rate. There are a number of reasons why this was expected. Laboratory confirmed cases are likely to represent a small proportion of cases in Somalia due to limited capacity to conduct tests, difficulty for people to access tests, and low access to health-care facilities. In addition, one-third of cases are thought to be asymptomatic and would be unaware of being infected, there may have been a low awareness of COVID-19 and the importance of testing, and the possibility of stigma may have discouraged those who were aware of the disease from seeking a test.

The syndromic score case rate also appeared to lag behind the confirmed case rate, with the decline following ‘wave one’ occurring some weeks after the decline in confirmed cases. This may be due to the concentration of laboratory testing in urban areas, where the initial rise in cases was seen. In contrast, the population included in the BRCiS monitoring system has a wider and more rural distribution, including remote areas where there may have been a delay in the rise and fall of transmission rates.

Repeated enumeration of all members of the sampled households allowed for the accurate monitoring of mortality within the cohort. Crude and under-five death rates rose during round 3 but remained below the emergency thresholds used in humanitarian contexts throughout the period reported here. This finding is supported by results from a study that used satellite monitoring of graveyards in and around Mogadishu to estimate total and excess mortality during the first wave of the pandemic [14]. Results from this work also suggest that SARS-CoV-2 had arrived in Somalia well before March 2020 as a rise in mortality was seen prior to the official reporting of the first case. Comparison with baseline mortality rates was not attempted in this current study due to the difficulty in establishing a baseline for our cohort population.

We chose the CRMS approach to estimate suspected COVID-19 deaths as it was based on previous, well established, verbal autopsy approaches, was made publicly available shortly after the onset of the pandemic, and was simple to use. Implementation of the questionnaire as an ODK form allowed for digital data capture and rapid collation and analysis of results.

Both the CRMS and the SSCD are new approaches that have not been validated using laboratory tests. Findings from other syndromic surveillance approaches have been published, such as the COVID Symptom Study in the UK, which uses self-reported data from app users, but in this context validation and ongoing adjustment of case identification algorithms has been possible due to the greater availability of testing and funding [15]. Work is also ongoing to validate the CRMS and other verbal autopsy approaches for identifying deaths due to COVID-19. Areas for further investigation are the estimated prior probabilities of signs and symptoms given a COVID-19 death or not and the unconditional probability of any death being COVID-related, which is likely to vary throughout the course of the pandemic and could be adjusted accordingly, as is currently done for HIV and malaria deaths in the full InterVA method on which CRMS is based [16]. Therefore, there remains a degree of uncertainty about both the sensitivity and specificity of the CRMS and the SSCD. However, the overall agreement between the trends in the case rates derived from laboratory test data and the SSCD, as well as the spatial pattern in cases observed as the pandemic progressed, supports the interim use of this approach until a formal validation is possible.

Decisions on how to interpret the CRMS-derived % probabilities for deaths from COVID-19 will influence the method’s sensitivity and specificity. Our sensitivity analysis shows little difference in results when using the population COVID-19-specific mortality fraction or the case-by-case 50% or 60% threshold cut-offs, with higher thresholds predictably leading to lower COVID-19 mortality estimates (see Supplementary file (web Figure W2)). Though absolute numbers and validity of COVID-19 mortality estimates will vary depending on the interpretation approach used, our overall description of patterns and trends would not have been changed by selection of an alternative threshold. Selection and consistent use of a single probability threshold provides significant advantages for monitoring COVID-19 over time and between settings.

The population included in the COVID-19 monitoring system was a convenience sample based on the geographic and socio-economic distribution of the pre-existing BRCiS cash transfer programme. Therefore, it does not offer a statistically representative picture of COVID-19 trends in Somalia as a whole, and results need to be interpreted with that in mind.

### Conclusions

Our experience argues that a flexible approach to data innovation is important during health emergencies and supports the need to keep approaches as simple as possible. To allow timely response, it may be necessary to build on pre-existing routine data collection systems and adapt to make them fit for purpose as threats emerge, rather than designing bespoke systems that may have many technical advantages but may not be useful if implementation is delayed. Approaches need to adapt to the availability of local resources, such as laboratory testing, and designs may need to aim for the best possible, rather than a technically perfect solution. However, constant assessment of the potential for bias in the data or analysis approach is essential.

Social safety net/welfare systems using e-transfers may provide useful platforms for ‘ready to go’ cohorts that can be utilised in response to rapid outbreaks or other disasters where telephone monitoring is appropriate, and the urgency of the situation does not allow for setting up new cohorts or data collection systems. However, data collection systems require support and investments in HIS now are an essential part of preparation for further outbreaks of COVID-19 and other future pandemics.

## Supplementary Material

Supplemental Material

## Capacity building & equity

Peter Byass often reflected on the very concept of ‘global health’ and strove to promote models of global health practice that addressed gaps in infrastructure, management, and human capital in LMICs. His well-used and much-stamped passports reflect his commitment to collaboration, partnerships and the importance of in-person relationships to build capacity. He was very conscious of and perhaps somewhat bewildered by academics from high-income countries ‘parachuting’ into low-income countries to set up systems or extract data with little or no effort to improve research capacity or to meaningfully engage with the people working and living there, who are undoubtedly better placed to define issues of importance and interpret data in relation to the on-the-ground context. Peter worked to amplify the local voice and build capacity for measurement and solutions to local problems. This recognised the fact that locally owned solutions are more likely to be sustainable, to have buy-in from critical stakeholders and, ultimately, to succeed in improving health.

The legacy of Peter’s ethos is apparent in our paper describing a pragmatic tool to understand the burden of COVID-19 in Somalia. The world’s ongoing failure to measure mortality, with the poorest populations remaining uncounted, is a matter of equity and basic human rights and it is of heightened importance during the current pandemic. We know poorer people are much less likely to have their deaths recorded, and this introduces a strong bias into health data. Our paper describes the rapid development and pragmatic adaptation of a locally run call-centre system of monitoring and evaluation built onto an existing social welfare scheme, which has allowed measurement among the poorest 10% of a community in a fragile country at the time of the COVID-19 pandemic. Our study population is likely to be under represented in hospital data or official COVID statistics and our innovative, equity-sensitive information systems – including the COVID-19 Rapid Mortality Surveillance tools on which Peter was working until his death – directly address such bias and inequity by giving this population a voice and ‘making them count’. Collaborative discussion and interpretation between academics, a non-governmental organisation, the Somali government and local communities have enabled meaningful use of these data and the initiative represents a commitment to local ownership and responsiveness to issues of global health importance.

## Paper context

Somalia has been affected by three waves of COVID-19 infections, but the case rate and cause-specific deaths can only be approximated due to low levels of testing. We estimated the symptomatic case and death rates due to COVID-19 using a syndromic score case definition and rapid verbal autopsy interviews. The suspected symptomatic case rate was approximately 99 times higher than the laboratory confirmed rate reported by WHO. This approach shows promise for monitoring the COVID-19 pandemic in low resource settings.
