# Supplementary material for: Data innovation in response to COVID-19 in Somalia: application of a syndromic case definition and rapid mortality assessment method
Source: Glob Health Action. 2022 Apr 4;14(Suppl):1983106. doi: 10.1080/16549716.2021.1983106 (PMC8986258; doi:10.1080/16549716.2021.1983106)
Supplement: Supplemental Material [file ZGHA_A_1983106_SM3885.docx]

**Web Figure 1.** Testing rate and test positivity in Somalia since the first identified case^1^


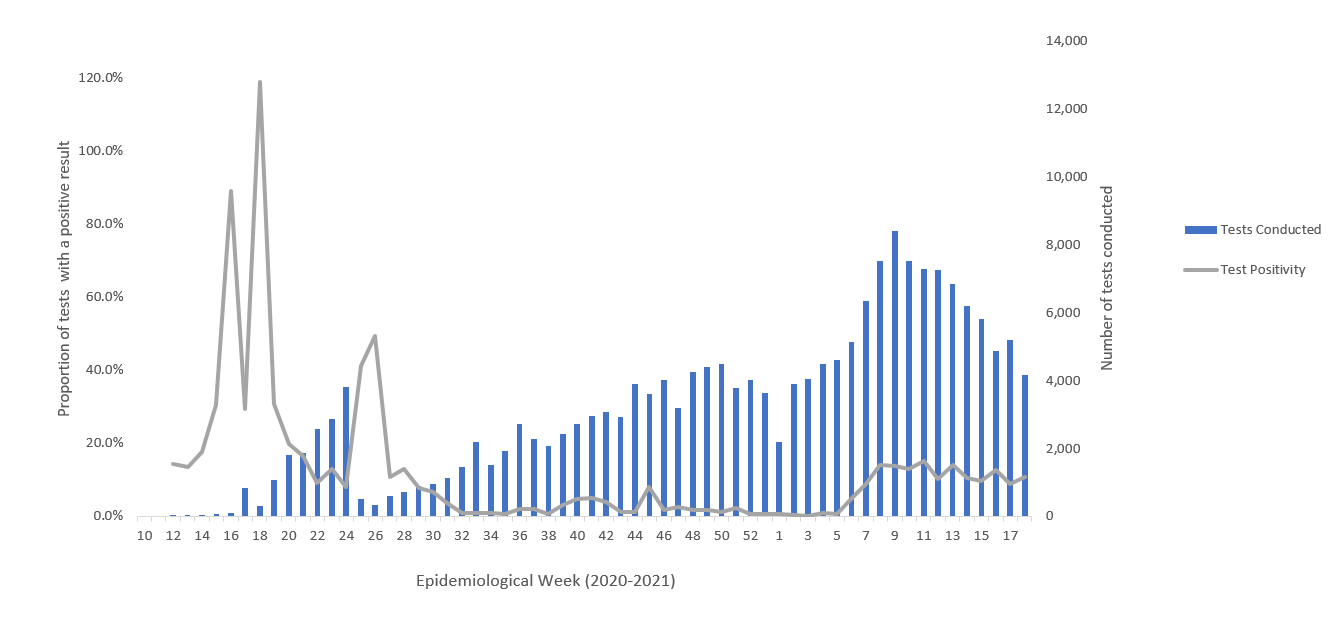


^1^WHO Somalia changed its reporting week start day from Saturday to Sunday in June 2020. In this figure we align all data with standard MMWR/CDC epi weeks using Sunday as the start date.

**Web Figure 2.** Comparison of different CRMS sensitivity thresholds on estimates of COVID-19 deaths (count, %, and rate)^1^


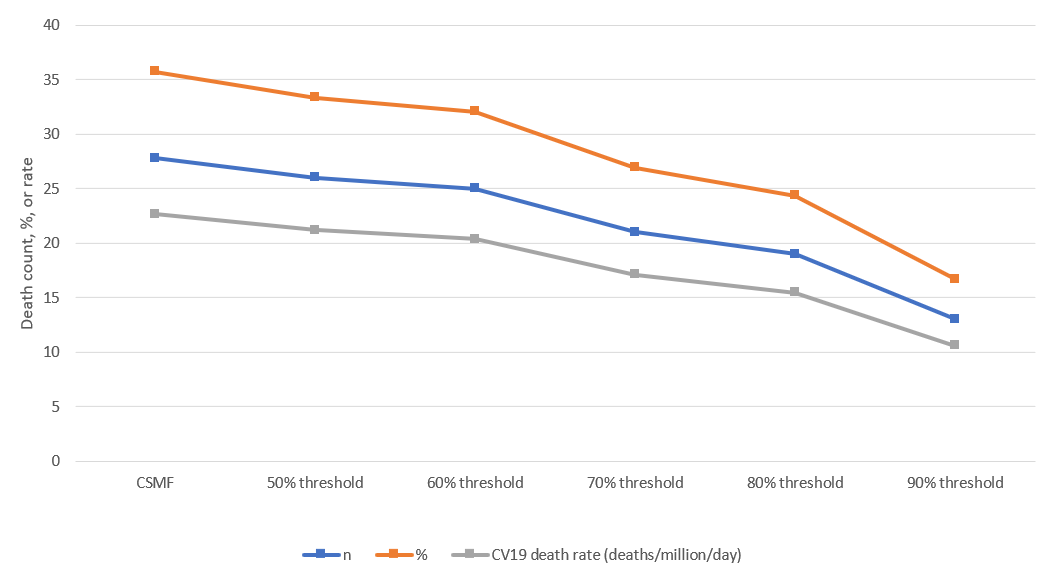


^1^ CRMS-derived percentage probabilities of individual deaths being due to COVID-19 are compared using the population COVID-specific mortality fractions (dividing the sum of the probability of COVID-19 among all deaths by the total number of deaths) and 10% incremental increases in the probability threshold from 50%, the threshold used in this paper, up to 90%.
